# Saffold Virus, a Human Theiler's-Like Cardiovirus, Is Ubiquitous and Causes Infection Early in Life

**DOI:** 10.1371/journal.ppat.1000416

**Published:** 2009-05-01

**Authors:** Jan Zoll, Sandra Erkens Hulshof, Kjerstin Lanke, Frans Verduyn Lunel, Willem J. G. Melchers, Esther Schoondermark-van de Ven, Merja Roivainen, Jochem M. D. Galama, Frank J. M. van Kuppeveld

**Affiliations:** 1 Department of Medical Microbiology, Virology Section, Radboud University Nijmegen Medical Centre, Nijmegen, The Netherlands; 2 QM Diagnostics BV. Radboud University Nijmegen Medical Centre, Nijmegen, The Netherlands; 3 Enterovirus Laboratory, National Public Health Institute, Helsinki, Finland; Stanford University School of Medicine, United States of America

## Abstract

The family *Picornaviridae* contains well-known human pathogens (e.g., poliovirus, coxsackievirus, rhinovirus, and parechovirus). In addition, this family contains a number of viruses that infect animals, including members of the genus *Cardiovirus* such as Encephalomyocarditis virus (EMCV) and Theiler's murine encephalomyelits virus (TMEV). The latter are important murine pathogens that cause myocarditis, type 1 diabetes and chronic inflammation in the brains, mimicking multiple sclerosis. Recently, a new picornavirus was isolated from humans, named Saffold virus (SAFV). The virus is genetically related to Theiler's virus and classified as a new species in the genus *Cardiovirus*, which until the discovery of SAFV did not contain human viruses. By analogy with the rodent cardioviruses, SAFV may be a relevant new human pathogen. Thus far, SAFVs have sporadically been detected by molecular techniques in respiratory and fecal specimens, but the epidemiology and clinical significance remained unclear. Here we describe the first cultivated SAFV type 3 (SAFV-3) isolate, its growth characteristics, full-length sequence, and epidemiology. Unlike the previously isolated SAFV-1 and -2 viruses, SAFV-3 showed efficient growth in several cell lines with a clear cytopathic effect. The latter allowed us to conduct a large-scale serological survey by a virus-neutralization assay. This survey showed that infection by SAFV-3 occurs early in life (>75% positive at 24 months) and that the seroprevalence reaches >90% in older children and adults. Neutralizing antibodies were found in serum samples collected in several countries in Europe, Africa, and Asia. In conclusion, this study describes the first cultivated SAFV-3 isolate, its full-length sequence, and epidemiology. SAFV-3 is a highly common and widespread human virus causing infection in early childhood. This finding has important implications for understanding the impact of these ubiquitous viruses and their possible role in acute and/or chronic disease.

## Author Summary

Recently, a new picornavirus was isolated from humans, named Saffold virus (SAFV). Picornaviruses are small RNA viruses with poliovirus as prototype. Saffold virus is genetically related to Theiler's virus, a member of the *Cardiovirus* genus, which until this recent discovery did not contain human viruses. Theiler's virus is an important mouse pathogen that causes chronic inflammation in the brains, closely resembling multiple sclerosis in humans. By analogy, SAFV may be a relevant human pathogen. Thus far, SAFVs have been sporadically detected by molecular techniques in respiratory and fecal specimens, but the epidemiology and clinical significance have remained unclear. Here we describe the first SAFV type 3 (SAFV-3) isolate, its growth characteristics in cell lines, full-length RNA-sequence, and epidemiology. Unlike the previously isolated SAFV-1 and SAFV-2, SAFV-3 grows well in cell lines, resulting in cell damage. This feature enabled us to conduct a large-scale serological survey for virus-neutralizing antibodies. This survey showed that SAFV-3 infection occurs early in life and that >90% of children >2 years and adults had antibodies. Neutralizing antibodies were found in serum samples collected in several countries in three continents. Hence, we concluded that SAFV-3 is a genuine and widespread human virus causing infection early in life.

## Introduction

Recent advances in molecular detection methods (*e.g.* viral oligonucleotide microarrays and viral metagenomics approaches) have led to the identification of many new viruses which are detected not only in symptomatic, but equally in individuals without any clinical manifestation. Insight into the potential role of these so-called “orphan” viruses in disease requires a detailed understanding of their genetic diversity and epidemiology. Classically, the association of an infectious agent with disease had to fulfill Koch's postulates, a concept that is no longer tenable in modern times. The clinical outcome of a virus infection may depend upon the conditions under which the infection is acquired: For example, poliomyelitis was seldom observed under conditions of poor sanitation, congenital rubella syndrome is a consequence of postponed childhood infection and some types of cancer are late events in which certain viruses play a crucial role. Moreover, it requires detailed insight in viral diversity, since it is well known that minor differences in the genetic make-up of viruses can cause major differences in their pathogenicity. The latter holds especially for RNA viruses such as the picornaviruses which due to their high mutation and recombination rates show remarkable genetic plasticity which may lead to serious pathology merely by accident [1].

The family *Picornaviridae* is one of the largest RNA virus families [reviewed in ref. 2], containing 8 established and 6 proposed genera and at almost 33 species. Four genera contain clinically important viruses infecting humans, i.e. *Enterovirus* (which since recently also includes the human rhinoviruses), *Parechovirus*, *Hepatovirus*, and *Kobuvirus*. This family also contains economically important animal pathogens such as *Foot-and-mouth-disease virus*, a species belonging to the *Aphthovirus* genus. Other well-known animal pathogens are *Theilovirus* and *Encephalomyocarditis virus* (EMCV), two species that belong to the *Cardiovirus* genus and are associated with disease in rodents and swine. The *Theilovirus* species is represented by Theiler's murine encephalomyelitis virus (TMEV) and rat encephalomyelitis virus (also called Theiler's rat virus, TRV). TMEV is an enteric pathogen that primarily causes asymptomatic infections of the alimentary tract. However, extra-intestinal infection can occur and produce acute fatal encephalomyelitis or a chronic demyelinating disease relevant for multiple sclerosis, depending on the TMEV strain involved [3]. TMEV can furthermore cause serious foetal pathology and placental damage, depending on the gestational phase of infection [4].

The question of whether authentic human cardioviruses exist has remained unclear for a long time. A Theiler's-like cardiovirus, named Vilyuisk human encephalomyelitis virus (VHEV), has been implicated in an outbreak of a neurodegenerative disease among the Yakuts people in Vilyuisk, Siberia, in the 1950s. However, this virus, which was isolated upon multiple passages in mice and cell cultures, shows close relationship with TRV and TMEV, raising the possibility that it represents a contaminating animal cardiovirus [5]. Recently, however, strong evidence has been obtained for the existence of human cardioviruses. In 2007, the genomic sequence was reported of a cardiovirus, provisionally named Saffold virus (SAFV), amplified in cell culture from the stool of an infant presenting with fever of unknown origin in 1981 [6]. Following this discovery, SAFVs were detected by molecular techniques in nasopharyngeal aspirates of 3 children with respiratory symptoms in Canada [7], in stool samples from 6 paediatric patients suffering from gastroenteritis from Germany and Brazil [8], and in 1 respiratory secretion from a patient presenting with an influenza-like illness and 6 stool specimens from patients in a gastroenteritis cohort in California [9]. Recently, SAFVs were detected in stool samples from 6 South Asian children with non-polio acute flaccid paralysis (AFP), but also in stool samples from 5 children without overt neurological symptoms [10]. All SAFVs identified to date are significantly divergent from the documented animal cardioviruses, especially in the region encoding the structural capsid proteins that are important for receptor binding. Phylogenetic analysis suggests the existence of 8 distinct genetic SAFV lineages [10],[11]. Together, these findings suggest that SAFVs are genuine human viruses that can be sporadically found in fecal and respiratory specimens of children, with or without symptomatology, but that until recently have gone largely undetected most likely because of their fastidious growth [6],[7]. Thus, their relationship with clinical disease remains to be established.

Here we report the first isolation, full-length sequence, characterization, and epidemiology of a SAFV-3 isolate. Evidence is provide that this SAFV is a ubiquitous human virus causing infection early in life. The implications of this finding for understanding its biology and possible associations with disease are discussed.

## Results

### Virus isolation and identification

In December 2007, a virus was isolated from a stool sample obtained from a 13 month-old boy who presented with vomiting. The sample was routinely sent in to exclude viral gastro-enteritis but the complaints were not due to an infection but to an anatomical mal-position of the jejunum. Culture on human embryonic lung fibroblasts (HELF) and tertiary monkey kidney cells (tMK) yielded a virus that was acid-stable, chloroform resistant and that by electron microscopy showed the size (approximately 30 nm) typical of a picornavirus (data not shown). However, the virus isolate could not be typed as an enterovirus or parechovirus.

To identify and characterize the unknown virus, which was provisionally named NL2007, the tMK isolate was propagated on HeLa cells and the virus was partially purified by centrifugation through a sucrose cushion. Nucleic acids were isolated from the pellet, reverse transcribed and amplified using a random PCR method. PCR fragments were cloned and sequenced. Based on a sequence comparison (BLASTn search), the virus was identified as a SAFV [11].

### Complete genome sequencing and phylogenetic analysis

A full genomic sequence was obtained by a genome walking method on amplified, partially overlapping fragments of 1 to 1.5 kb. The sequence of the end of the 5′ UTR was determined using a 5′-RACE amplification protocol. The genome contained a 5′ UTR of 1,042 nucleotides (nt), an open reading frame (ORF) of 6,888 nt, and a 3′ UTR of 121 nt followed by a poly(A) tail (GenBank accession number FM207487).

**Figure 1:**
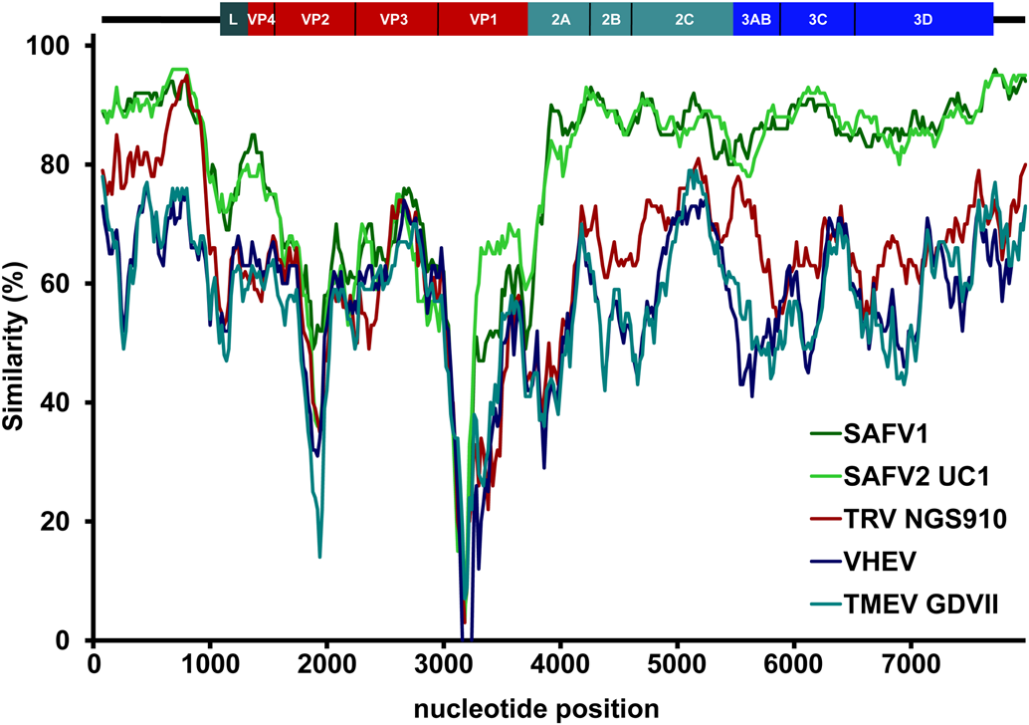
Similarity plot analysis based on complete cardiovirus sequences using SAFV-3 (NL2007) as query sequence. Analysis made use of a sliding window of 200 bases and a step size of 20 bases. The y-axis shows the percentage of similarity between the selected cardiovirus sequences and the query sequence.

Alignments and phylogenetic analyses were performed with available SAFV sequences (which do not include those of the recently identified SAFV-4 to -8 genotypes [10], which have not yet been published). Alignment of the sequence of isolate NL2007 with other full-length cardiovirus sequences showed high sequence similarity with SAFV-1 [6] and SAFV-2 (UC1) [9] in the 5′UTR, the regions encoding the nonstructural proteins (i.e. the leader protein and the P2 and P3 region proteins), and the 3′UTR. Less similarity was observed in the capsid coding P1 region (**Fig. 1**). The overall nt identity with TMEV, TRV, and VHEV was considerably lower.

**Figure 2:**
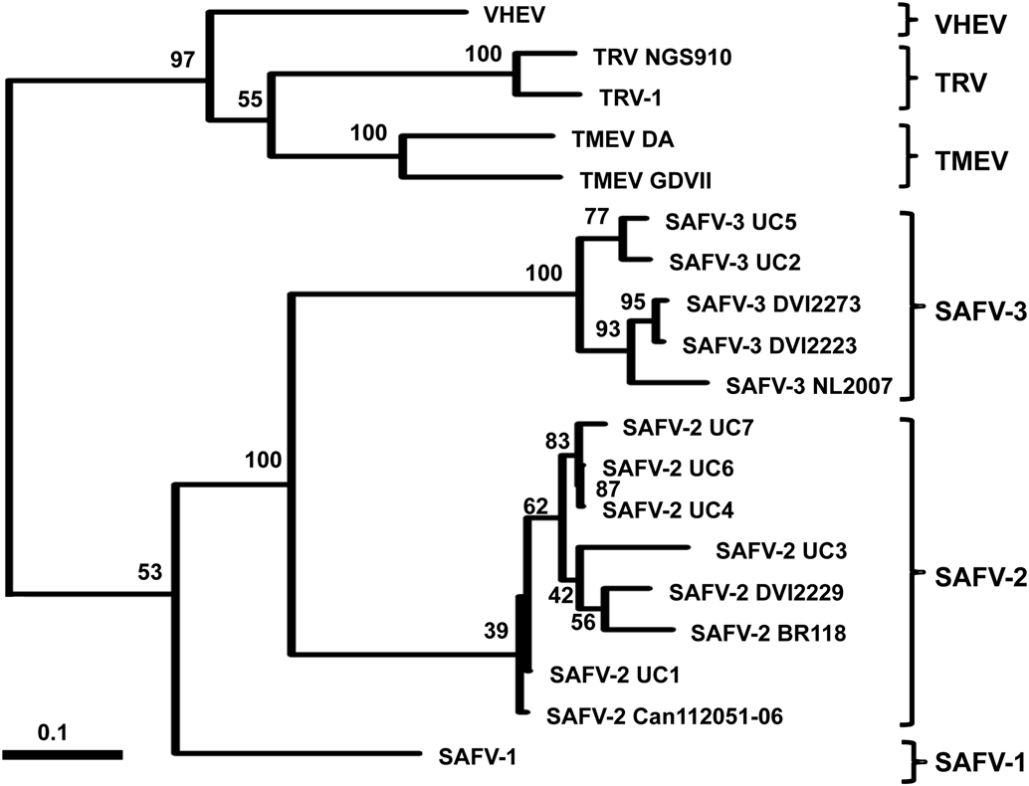
Phylogenetic tree based on cardiovirus VP1 sequences. Sequences were analyzed as indicated in the text. Bootstrap values from 100 replicate trees are shown next to the branches. The bars represent the number of substitutions per site.

Phylogenetic analysis of the available VP1 coding regions revealed six clades: TMEV, TRV, VHEV, SAFV-1, SAFV-2, and SAFV-3 (**Fig. 2**). Similar clades were recovered upon phylogenetic analysis of the available P1 protein sequences (data not shown). Our NL2007 strain represents a new member of the SAFV-3 lineage, to date containing only P1 sequences of 2 German strains [8] and VP1 sequences of 2 American strains [9]. Herewith, NL 2007 is the first viable SAFV-3 reported and the sequence is the first complete SAFV-3 genomic sequence: SAFV-3(NL2007).

### Molecular characteristics of SAFV-3(NL2007) and comparison with other cardioviruses

The genetic organization of SAFV-3(NL2007) is similar to that of the other cardioviruses [reviewed in ref. 9]. The nt and amino acid (aa) identities of the UTRs and the individual viral proteins with other Theiloviruses are shown in **Table S1**.

The 5′ and 3′ UTRs of SAFV-3(NL-2007) are highly conserved to those of SAFV-1 and -2 (90 and 97% nt identity, respectively). The 5′ UTR contains a type II IRES typical for the cardioviruses. Secondary structures of the RNA replication elements in the UTRs, as predicted by MFOLD [12], resemble those in the other Theiloviruses (data not shown). All picornaviruses contain an additional higher-order RNA structure, the *cis*-acting replication element (CRE), involved in uridylylation of the peptide primer, VPg [13]. TMEV contains a CRE within the VP2 encoding region [14]. Alignment of the available cardiovirus sequences revealed the presence of a CRE within the same region of the SAFVs (**Fig. S1**). RNA structure prediction showed a structural element similar to that of TMEV [14].

The leader (L) protein, an interferon antagonist [15],[16], is the least conserved non-structural protein (78–80% nt identity, 82–83% aa identity). However, the typical characteristics of the cardiovirus L protein - i.e. a zinc-finger motif, an acidic region and a serine/threonine-rich domain - are well conserved (**Fig. S2A**). As in SAFV-1 and -2, the serine/threonine-rich domain is partially deleted. TMEV also contains an alternative open reading frame (ORF) encoding L* [15], a 156 aa protein associated with neurovirulence and persistent infection [17],[18]. The corresponding alternative ORF in SAFV-1 and -2 encodes severely truncated L* proteins of only 57 aa and 34 aa, respectively. SAFV-3, like SAFV-2, contains a truncated L* protein of only 34 aa (**Fig. S2B**). Whether the SAFV L* proteins are produced and have any function remains to be shown.

The overall nt identity in the P2 and P3 regions to SAFV-1 and -2 is 82–90%. At the aa level, the P2 and P3 region proteins in the SAFVs are even 95–100% identical. The corresponding regions of TMEV, TRV, and VHEV were more divergent (61–90% identity at aa level) , although the P2 and P3 region proteins of SAFVs were more closely related to those of TRV than to those of TMEV and VHEV. In contrast to the high similarity for P2 and P3, SAFV-3(NL2007) differs significantly from SAFV-1 and SAFV-2 in the P1 region. The divergence is highest in VP1 (64–69% nt identity, 68–73% aa identity) and VP2 (71–72% nt identity, 77–79% aa identity) and lowest in VP4 region (82–84% nt identity, 99–100% aa identity), which is located at the interior of the capsid. The identity of SAFV-3(NL2007) with the Germany SAFV-3 strains (2223 and 2273) is 91% (nt) and 98% (aa), whereas that with the US SAFV-3 strains (UC1 and UC5) is 86% (nt) and 97% (aa).

**Figure 3:**
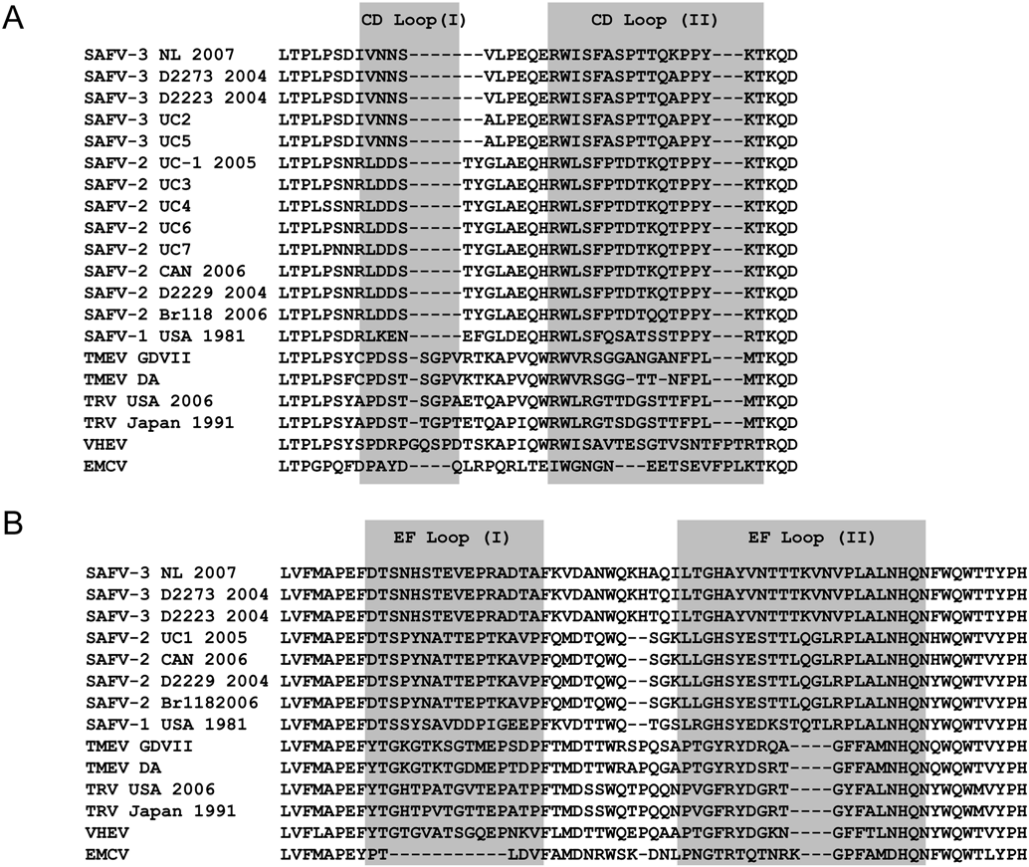
Alignment of cardiovirus VP1 CD (A) and VP2 EF (B) loop sequences. Loop sequences are boxed (grey). SAFV-3(NL2007) amino acids 270–338 (VP2) and 716–753(VP1) are shown.

Cardioviruses contain 4 small loops at their outer surface that are important sites for recognition by neutralizing antibodies [19]. Two loops are part of the VP1 CD loop structure and 2 are part of the VP2 EF loop structure. These loop sequences, which are the regions of greatest divergence, are unique for each of the different SAFVs and well-conserved among different strains belonging to a specific SAFV genotype. Comparison of the CD and EF loop structures confirmed the close relationship of the NL2007 virus with the 2 German and the 2 US SAFV-3 sequences (**Fig. 3**). Remarkably, SAFV-3(NL2007) contains a unique aa substitution (lysine instead of alanine) in VP1 CD loop-II.

### Virus replication characteristics

**Figure 4:**
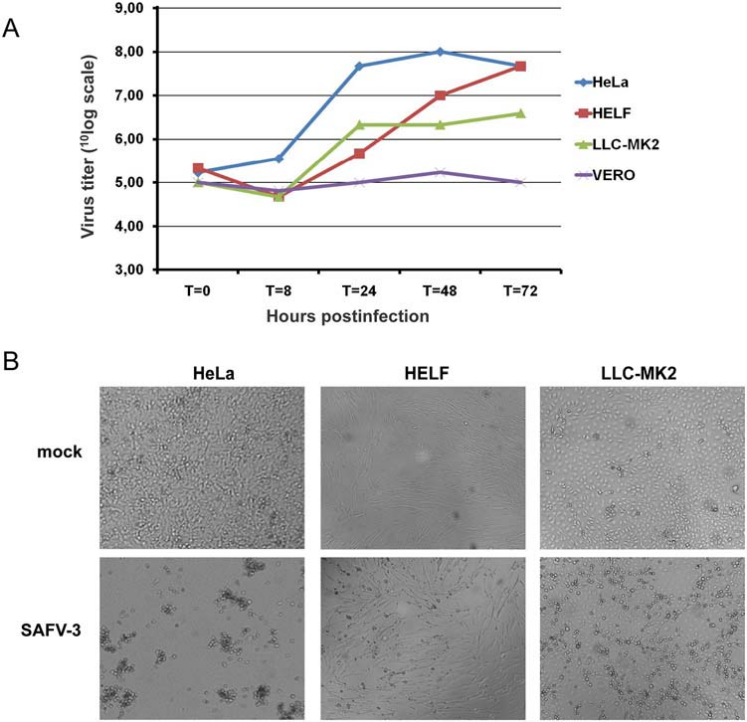
Replication of SAFV-3(NL2007) in cell lines. (A) Growth kinetics of SAFV-3(NL2007) on different cell types. Cells were infected with SAFV-3(NL2007) at a multiplicity of infection of 10. At the indicated times, cells were subjected to three cycles of freezing and thawing, and the virus titers were determined by endpoint titration on HeLa cells. (B) Cytopathic effect in the indicated cell lines at 3 days post infection with SAFV-3(NL2007).

Thus far, SAFVs have been shown difficult to propagate on cell lines. Only two SAFVs have been propagated in cell culture, a SAFV-1 in human fetal diploid kidney (HFDK) cells [6] and a SAFV-2 in rhesus monkey kidney cells (LLC-MK2) [7]. Both viruses were reported unable to replicate on many other cell lines. As described above, the SAFV-3(NL2007) virus was isolated on HELF and tMK cells and passaged on HeLa cells (a human epithelial cervical carcinoma cell line), on which the virus produced a rapid cytopathic effect (CPE) culminating in complete lysis of the cell monolayer. Additional studies showed replication - as tested by end-point titration on HeLa cells - of SAFV-3(NL2007) in rhesus monkey kidney cells (LLC-MK2), but not in human embryonic kidney (HEK293), African green monkey kidney (Vero), Buffalo green monkey kidney (BGMK), or Madin-Darby canine kidney (MDCK) cells. Viral growth curves and CPE produced on HeLa, HELF, and LLC-MK2 cells are shown in **Fig. 4A** and **Fig. 4B**, respectively. The data show that virus replication was most efficient in HeLa cells resulting in a ∼1000-fold increase in virus titer within 24 hours and complete lysis of the cell monolayer in 2–3 days.

### Prevalence of SAFV-3 antibodies

**Figure 5:**
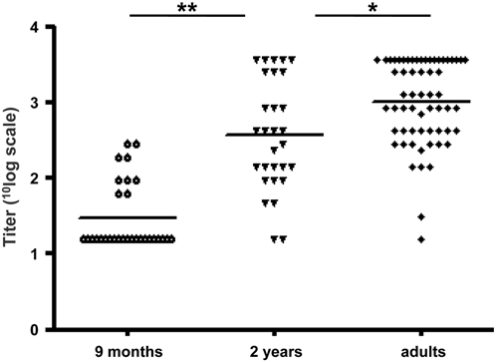
Antibody prevalence and geometric mean titers of neutralizing antibodies against SAFV-3 in sera of 30 children 9 months of age, 30 children at the age of 2 years and 60 adult women at week 12 of pregnancy, all from the Netherlands. *, *p*<0.05; **, *p*<0.0005.

Growth properties of SAFV-3(NL2007) with a clear CPE in HeLa cells made it possible to conduct a serological survey by virus neutralization (VN). A total of 210 serum samples sent to our laboratory for routine diagnostics, were tested in the VN assay. These included serum samples from 60 adults (females at week 12 of pregnancy) collected in 2004 and serum samples from 120 paediatric patients, of different age groups: 2, 4, 8 and 10 year of age, 30 samples each, collected in 2006–2007. In addition, serum samples obtained in 2006–2007 from 30 infants, 9 months of age, were tested. At this age, serum IgG levels start to rise from a physiological nadir and remaining maternal antibody levels are low. As is shown in **Fig. 5**, 21/30 infants tested negative (titers <1∶10) and geometric mean titers of neutralizing antibodies were lowest at the age of 9 months. Titers rose sharply between the age of 9 months and of 2 years (*p*<0.0005, unpaired t-test, two-tailed). Of 90 children between 4–10 years of age, 92% had neutralizing antibodies against SAFV-3 and 98% of 60 adults (**Fig. 5**, **Table 1**). Titers of neutralizing antibodies in the adults were higher than those in the 2-year old children (*p*<0.05) (**Fig. 5**). None of the 30 serum samples from the 2-year-old children contained antibodies against EMCV, illustrating the specificity of the VN assay. These results indicate that antibodies against SAFV-3 are highly prevalent in the Netherlands and that infection most likely occurs early in life.

**Table 1:** Prevalence of neutralizing antibodies against SAFV-3 in sera from infants, young children and adults from different countries.

Country	# Samples	Age range	Period^1^	# Positive (%)^2^
Netherlands	30	9 months	2006/2007	9 (30)
Netherlands	30	24 months	2006/2007	28 (93)
Netherlands	90	4, 8,10 yrs	2006/2007	83 (92)
Netherlands	60	18–39 yrs	2004	59 (98)
Finland	30	2–2.5 yrs	1997/1998	23 (77)
Cameroon	29	5–15 yrs	1997	29 (100)
Mali	43	10–40 yrs	2006	43 (100)
Indonesia (Java)	30	6–40 yrs	1997/1998	30 (100)
Indonesia (Sumba)	33	5–40 yrs	2007	33 (100)

1 period, blood samples were collected.

2 titer≥10.

Next, we investigated the prevalence of SAFV-3 antibodies in other countries. Serum samples from 30 children from Finland (age 24–30 months), collected in 1997–1998, were tested. Furthermore, we tested serum samples from 72 African individuals (from Mali and from Cameroon, collected in 2006 and 1997 respectively) and from 63 Indonesian individuals (from Java and from Sumba, collected in 1997 and 2007 respectively). Neutralizing antibodies were found in 77% of Finnish children and 100% of people living in Africa or Indonesia (**Table 1**), indicating that infection with SAFV-3 has a wide spread distribution among humans. Moreover, the presence of neutralizing antibodies in samples collected in 1997–1998 proves that this particular virus or a closely related one is circulating for at least 10 years.

We also investigated the possible occurrence of SAFV-3 antibodies in blood specimens from laboratory rodents. For this purpose, we used pooled serum samples from rats, mice, hamsters, guinea pigs, and rabbits. No antibodies were observed in the samples from either of these rodent species. In addition, we tested pooled serum samples from rats and mice containing high titer antibodies to TMEV or EMCV. Again, no SAFV-3 neutralizing antibodies were detected (data not shown). Together, these data suggest that it is unlikely that SAFV-3 is a common virus in rodents. Moreover, these data further emphasize the specificity of our neutralization assay.

## Discussion

The family Picornaviridae contains a large number of notorious human and animal pathogens. The recent identification of human cardioviruses, Saffold viruses, has prompted studies to establish an association between these viruses and disease. Current information on SAFVs is almost exclusively based on molecular detection of these viruses in respiratory and stool samples, where virus is found at low frequency and mainly in children under 6 years of age [7],[8],[9], but the clinical significance of SAFV infections is unclear. From current data as well as from unpublished results by ourselves and others, it appears that further molecular surveys on respiratory and stool specimens may not readily provide an answer. For gaining more insight into the significance of SAFVs, complementary studies are required, such as (i) serological surveys to address the prevalence of infection, (ii) a detailed scrutiny for signs of systemic infection (testing in blood, biopsies, etc), and (iii) analysis of the possible implications of these viruses in terms of ecology and integrity of the gut as major immunological border of host defense. Here, the first serological survey of a SAFV is presented and the possible impact of these viruses is discussed.

The serological survey was performed with a SAFV-3 isolate that we isolated and characterized. Our SAFV-3 isolate is the first reported SAFV that efficiently replicates in cell lines and produces a clear cytopathic effect, thereby allowing to conduct a large-scale survey by means of a specific virus-neutralization assay. Using the assay, we showed that SAFV-3 is a highly common and wide-spread human virus causing infection in early childhood: the seroprevalence of SAFV-3 reaches high levels (>90%) in young children and adults. Nine months after birth, maternal antibodies have almost disappeared. In accordance, at nine months of age, many infants that we tested were seronegative and those that had antibody showed low titers which may still have been of maternal origin. A steep rise was found in 2-year-old children, both in seroprevalence and in titers, which indicates that infection occurs frequently and early in life. It is too early to state that SAFV infections occur before the age of 2 years because age of infection can be influenced by periodicity of outbreaks with a particular genotype (e.g. SAFV-3). The latter could explain why the Finnish children in 1997/98 showed a somewhat lower seroprevalence. Alternatively, the low population density in Finland compared to the Netherlands may have limited the speed of circulation of the virus. High rates of SAFV-3 antibody were furthermore found in African countries and in Indonesia, strongly supporting that SAFVs are ubiquitous human viruses with a global prevalence: SAFVs have now been reported from countries in North- and South-America, Europe, South-Asia and Africa [6]–[10, this study].

A high seroprevalence contrasts with the low numbers (<1%) of respiratory and fecal samples that tested positive by PCR, at least in developed countries [6],[7]. We have no explanation for this discrepancy. A possible explanation can be that virus is produced and excreted only for a very brief period, limiting the chance of its detection. However, the latter would also shorten the period of contagiousness and virus transmission which is not in line with ubiquity and infection early in life. The majority of SAFVs has been detected in feces suggesting transmission by the fecal-oral route. Fecal-oral transmission is generally associated with a prolonged fecal excretion typically found for enteroviruses, parechoviruses as well as rodent cardioviruses. Enteroviruses and parechoviruses are detected 5–10 times more frequently in feces compared to SAFVs in developed countries [20, our unpublished data] although their epidemiology is quite similar. A higher percentage of SAFVs (10% positive feces samples) was recently reported in children from Pakistan and Afghanistan [10]. This may be due to differences in sanitation, but this remains to be established.

Another aspect of consideration is the specificity of serology. Virus neutralization tests are highly specific and can discriminate serotypes within a single species [21]. From the phylogenetic analysis in this study it is unlikely that the neutralization assay with SAFV-3(NL2007) measured antibodies that cross-react with other genotypes, let it be with other species: The phylogenetic distinction between SAFV genotypes is largely based on differences within the capsid region. Known SAFV-3 strains show a high level of conservation (cave drift) in their VP1 protein (97–98% aa identity) but a significant level of divergence with that of SAFV-1 and -2 (68–73% aa identity), which is most probably due to immune selective pressure. The reported divergence is analogous to that observed in VP1 between serotypes of human enteroviruses, where nt differences in VP1 of more than 25% or aa differences of more than 12% are indicative of different serotypes [22]–[24]. The highest variability among the different SAFVs was observed in the VP1 CD loop and the VP2 EF loop which are located at the outer surface of the virion and implicated as important sites for binding of neutralizing antibodies. Given the large differences between their VP1 proteins, it is reasonable to assume that the 8 SAFV genotypes identified to date are serologically distinct. Accepting that the serological data in this study regard largely, if not entirely, one single type (SAFV-3), the contrast with sparse detection of SAFVs by PCR is even greater and additional studies are required to solve this discrepancy.

Our serological data are consistent with infection early in life which is corroborated by PCR data being mainly positive in children below 6 years of age [6]–[9]. In this age group, SAFVs may be a cause of respiratory disease [7],[9] and/or gastro-enteritis [8],[9], but the evidence is thin and the possibility of it being an innocent bystander in fecal samples is not excluded. Similarly, the relevance of SAFVs in the feces of non-polio AFP cases in South Asia remains to be established since SAFVs were also found in asymptomatic children [10]. Apparently, severe pathology due to SAFV infection is rare, at least in the young, and whether it occurs remains to be established. Importantly, not all adults that were tested in our serological study have passed SAFV-3 infection and a small proportion of them may thus still be susceptible (as shown in **Fig. 5** for pregnant women). Moreover, a high seroprevalence early in life causes a severe constraint on further circulation of the virus, which can be overcome by subtle antigenic drift, as known for influenza viruses, resulting in re-infection at older age. Variation in the loop sequences - as observed in the SAFV-3(NL2007) isolate, which differs a single aa from other SAFV-3 strains in one of its VP1 loops (**Fig. 3**) - is in agreement with such a drift, but whether re-infections occur remains to be proven: Significantly higher antibody levels in adults, compared to children (**Fig. 5**), may suggest so but an increase in antibody levels may also be the consequence of infection by other members of the species (phenomenon of the original antigenic sin, OAS) [25]. Thus, one has to consider that adults may still be susceptible and may acquire infection under unfavorable conditions, e.g. during pregnancy when infection may be a threat for the fetus or newborn [4],[26],[27].

Although enteroviruses and parechoviruses mostly cause asymptomatic infections, they can cause severe pathology depending on the conditions of infection, which involve both host determinants (e.g. age of infection, genetic background) and virus determinants (e.g., serotype, virulence). Some good examples are provided by the *human enterovirus C* (HEV-C) species which includes coxsackie A viruses causing mild respiratory symptoms, as well as the polioviruses which can cause severe neurological disease and paralysis [2]. Virulence of a virus variant may depend on a single or a few amino acid substitutions as is illustrated by poliovirus type 3 Sabin, the vaccine strain, which differs from the parental strain in not more than 3 aa [28]. Similarly, SAFV as species may comprise a whole spectrum of variants, including variants that cause pathology dependent on the conditions of infection and the virus type and/or strain involved. For the two animal species of Cardiovirus there is robust evidence that such variations exists. A single point mutation, resulting in a change in amino acid within the VP1 capsid region, is sufficient to transform EMCV from wild type into a diabetogenic phenotype in mice [29]. TMEV strains are divided into two sublineages according to the pathology they cause in the central nervous system of susceptible mice. Highly neurovirulent strains (e.g. GDVII) cause an acute and lethal encephalomyelitis. Low neurovirulent strains (e.g. DA) cause a mild, transient encephalitis that is followed by viral persistence in the spinal cord white matter, resulting in a chronic inflammatory response and lesions of primary demyelination, highly similar to the signs and symptoms observed in Multiple Sclerosis [2],[3]. The difference in pathology caused by these TMEV strains reflects most likely a difference in neural tropism in the brains due to a difference in coreceptor usage [30]. Different SAFVs may also exhibit a difference in tropism. Consistent with this, we found a difference in tropism between our SAFV-3 isolate and a SAFV-2 that was isolated in our lab. This latter virus could be propagated on LLC-MK2 cells, albeit poorly, consistent with the growth pattern of SAFV-2 Can112051 on this cell type [7], but failed to replicate on HeLa or HELF cells (data not shown). The intriguing possibility of involvement of SAFV in MS awaits further investigation. The search for implications of SAFV infections is just starting and as depicted before, it may require testing for systemic infection, e.g. in blood, cerebrospinal fluid or biopsies of affected organs (e.g. brains). Results for cerebrospinal fluid specimens have been reported, but so far with a negative outcome [9]. Hence, the human cardioviruses are currently orphan viruses as were ECHO viruses in the past.

New molecular approaches have rapidly expanded the list of enteric viruses (e.g. SAFV, Cosa virus, Boca virus) for which pathology is currently less evident [31],[32]. Moreover, the increased sensitivity of nucleic acid-based techniques confronts the physician with a relatively new phenomenon of co-infection with 2, 3, or even more viruses at a time, making it hard to predict which of these is the culprit, if causing pathology at all. The latter holds not only for these new viruses but also for well known pathogens as enterovirus, parechovirus and adenovirus. Herewith, a picture emerges of a ‘viral flora’ quite similar to the microbiome of intestinal resident bacteria which are largely beneficial to the host, e.g. by competing out pathogenic invaders and playing an active role in shaping an intestinal immune barrier [33],[34]. A beneficial effect (cross-protection against bacterial invaders) has also been demonstrated for beta- and gamma-herpesviruses in mice [35]. Human cytomegalovirus has probably a similar effect as it changes the T-cell system dramatically, inducing a unique population of effector-memory CD8 T cells with innate-response features [36],[37]. Viruses, however, are -by nature- host-cell invasive microbes (which gut-resident bacteria are not) and a beneficial role of enteric viruses has not been investigated. Coxsackie B viruses, and probably also other enteric viruses, affect the tight junctions of the intestinal epithelial cells, which are the gatekeepers of the intestine, thereby increasing gut leakiness [38]. The latter is a direct cause of local inflammation, it may alter mucosal immunity in a beneficial way but leakiness has also been associated with chronic inflammatory disease, as type 1 diabetes and celiac disease [39]. Several lines of evidence indicate a role for coxsackie B viruses and other enteric viruses in type 1 diabetes in humans [Bibr ppat.1000416-Hyoty1] [reviewed in 40 and 41]. A possible role of SAFV in the development of this disease should be considered in future studies. In such studies, a role of different genotypes and, by analogy with EMCV, different strains belonging to one genotype should be taken into account.

In conclusion, the SAFVs, are human viruses belonging to the genus *Cardiovirus* of the picornavirus family. SAFV-3, one of its genotypes, shows a high seroprevalence on three different continents, indicating wide-spread presence of infection among humans. Infection by SAFV-3 is most likely acquired early in life. Infection may pass without symptoms but the full clinical and/or biological spectrum remains still to be established.

## Materials and Methods

### Viral culture and characterisation

Fecal and respiratory samples are routinely cultured on human embryonic lung fibroblasts (HELF), tertiary monkey kidney cells (tMK) and Hep-2 cells. Isolates with a cytopathic effect (CPE) suggestive for a picornavirus are tested for acid- and chloroform resistance. If so, the isolates are typed using enterovirus typing serum pools provided by the National Institute for Public Health and Environment, Bilthoven, The Netherlands (RIVM) [42]. When typing with serum pools provides no answer, the isolate is genetically typed by PCR with primers specific for enterovirus, parechovirus and rhinovirus.

### Complete genome cloning and sequencing

The virus was partially purified by centrifugation through a layer of 30% (w/w) sucrose in PBS for 3 h at 250,000×G, 4°C. RNA was isolated from the pellet using Trizol-B (Invitrogen) according to the protocol of the manufacturer and amplified using a random PCR method [43]. Reverse transcription was performed with Transcriptor RT (Roche) and subsequent PCR using the long range PCR kit (Roche) according to the protocols of the manufacturer. PCR fragments were cloned into the pTOPO vector (Invitrogen) and sequenced. Based on a sequence comparison (BLASTn search), the virus was identified as a SAFV. A full genomic sequence was obtained by a genome walking method on amplified, partially overlapping fragments of 1 to 1.5 kb. The 5′UTR sequences were determined using the 5′-RACE System (Invitrogen) according to the manufacturer's protocol.

### Phylogenetic analysis

Nucleotide and protein sequences of the following cardiovirus genomes were obtained from GenBank (accession numbers): TRV NGS910 Japan 1991 (AB090161); TRV-1 USA 2006 (EU815052); VHEV (M80888 and EU723237); TMEV DA (M20301); TMEV GDVII (NC_001366); SAFV-1 USA 1981 (NC_009448); SAFV-2 D2229 2004 (EU681176); SAFV-2 UC-1 2005 (EU376394); SAFV-2 Can 2006 (AM922293); SAFV-2 BR118 2006 (EU681177); SAFV-3 D2273 2004 (EU681178); SAFV-3 D2223 2004 (EU681179); SAFV-3 NL2007 (FM207487). Sequence alignment was performed using ClustalW [44]. Phylogenetic trees were constructed by Maximum Likelihood algorithm as implemented in the Phylip software package version 3.67 [45]. Similarity plots were made using SimPlot version 3.5.1 [46].

### Growth curves

A virus stock of SAFV-3 was produced on HeLa cells, aliquoted, and titrated by end-point titration on HeLa cells. For growth curves, subconfluent monolayer cells were infected with virus at a multiplicity of infection (MOI) of 10 for 30 min at room temperature. Cells were grown at 36°C for 0, 8, 24, 48 or 72 h. At these time points, viruses were released by three cycles of freezing and thawing. Virus titers were determined by end-point titration on HeLa cells as previously described [47].

### Virus neutralisation assay

Virus neutralisation (VN) assays were performed on human epithelial cervical carcinoma (HeLa) cells in a microtiter assay as described for enteroviruses [48]. Six three-fold serum-dilution steps were used, starting at a 1∶10 dilution. The highest dilution that completely inhibited viral CPE was taken as titer. Next to SAFV, an EMCV (strain Mengovirus) was tested for comparison.

### Serum samples for virus neutralisation assay

A total of 375 serum specimens were tested, belonging to three categories: a) routine viral diagnostic samples (n = 210); b) serum samples from 30 Finnish children collected for a surveillance study on antibodies against polioviruses; c) samples from 135 individuals living in two African countries and two different areas in Indonesia collected for studies on malaria. Permission for anonymous testing of the samples fulfilled the terms of the ethical code “Goed Gebruik” of the Netherlands Federation of Medical Scientific Societies (Federa).

Rodent serum samples have been provided by QM Diagnostics BV. Pooled rodent sera have been used which were tested negative for a viral annual test panel recommended by FELASA (Federation of the European Laboratory Animal Science Association). Sera were derived from rats, mice, hamsters, guinea pigs, and rabbits. In addition, pooled sera from rats positive for TMEV and sera from mice containing high titer antibodies to TMEV or EMCV confirmed positive by ELISA and immunoblot were tested.

## Supporting Information

Figure S1 Structure and alignment of SAFV CRE. (A) Secondary structure of the SAFV-3(NL2007) CRE as predicted by MFOLD. (B) Alignment of cardiovirus VP2 sequences (nt 1533–1569, numbering according to SAFV-3(NL-2007)) corresponding to the CRE region. Nucleotides involved in stem formation are indicated.
(0.07 MB PDF)

Figure S2 SAFV L and L* proteins. (A) Sequence alignment of the cardiovirus leader proteins. Cysteine and histidine residues involved in the formation of the zinc-finger are shaded. The zinc-finger-, acidic-, and the serine/threonine-rich domains are indicated. The EMCV L protein is phosphorylated at threonine-47. This phosphorylation site fails in TMEV and VHEV. It is importantly to note that although all SAFVs contain a threonine at the corresponding position, the identity of the surrounding aa render it an unfavorable phosphorylation site. (B) Sequence alignment of putative cardiovirus L* proteins. The first 75 aa from the +1 frame shifted ORF are shown. Stop codons are depicted as *. Note that the L* proteins of the TMEV-GDVII strain and the SAFVs contain an ACG start codon.
(0.07 MB PDF)

Table S1 Nucleotide and amino acid (in parenthesis) identity of SAFV-3(NL2007) UTRs and proteins to other Theiloviruses from which full-length sequences or polyprotein coding regions are known.
(0.09 MB PDF)
