# Supplementary material for: Saffold Virus, a Human Theiler's-Like Cardiovirus, Is Ubiquitous and Causes Infection Early in Life
Source: PLoS Pathog. 2009 May 1;5(5):e1000416. doi: 10.1371/journal.ppat.1000416 (PMC2670511; doi:10.1371/journal.ppat.1000416)
Supplement: Figure S1 — Structure and alignment of SAFV CRE. (A) Secondary structure of the SAFV-3(NL2007) CRE as predicted by MFOLD. (B) Alignment of cardiovirus VP2 sequences (nt 1533–1569, numbering according to SAFV-3(NL-2007)) corresponding to the CRE region. Nucleotides involved in stem formation are indicated. (0.07 MB PDF) [file ppat.1000416.s001.pdf]

A

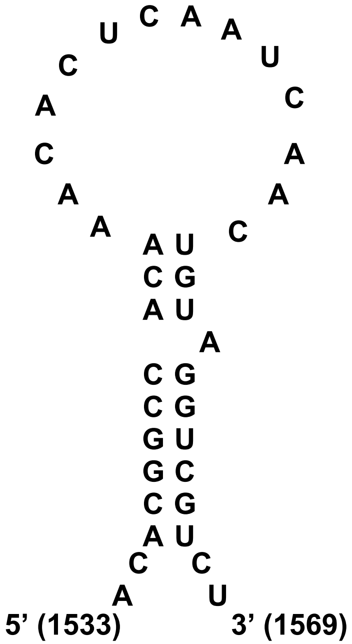

B

```
SAFV-3 NL2007      ACACGGCCACAAACACUCAAUCAACUGUAGGUCGUCU
SAFV-3 DVI2223     .....................................
SAFV-3 DVI2273     .....................................
SAFV-2 UC1         ......................U..A.....C...U.
SAFV-2 Can112051   ......................U..G.....C...U.
SAFV-2 BR118       ......................C..A.....C...U.
SAFV-2 DVI2229     .U....................C..A.........U.
SAFV-1             .......U..............C..A..G......U.
TMEV GDVII         .UU.............A..G..U.....U..C..G..
TMEV DA            .UU.............C.....U.....U.....A..
TRV-1              .UU.....GU............GU.A.....C..A..
TRV NGS910         .UU.....GU......C.......U.A.....C..A..
VHEV               .UU...................CU.C..U..C..G..
EMCV               ......U.........C........C..U........
                     stem                     stem
```
