# Supplementary material for: Saffold Virus, a Human Theiler's-Like Cardiovirus, Is Ubiquitous and Causes Infection Early in Life
Source: PLoS Pathog. 2009 May 1;5(5):e1000416. doi: 10.1371/journal.ppat.1000416 (PMC2670511; doi:10.1371/journal.ppat.1000416)
Supplement: Figure S2 — SAFV L and L* proteins. (A) Sequence alignment of the cardiovirus leader proteins. Cysteine and histidine residues involved in the formation of the zinc-finger are shaded. The zinc-finger-, acidic-, and the serine/threonine-rich domains are indicated. The EMCV L protein is phosphorylated at threonine-47. This phosphorylation site fails in TMEV and VHEV. It is importantly to note that although all SAFVs contain a threonine at the corresponding position, the identity of the surrounding aa render it an unfavorable phosphorylation site. (B) Sequence alignment of putative cardiovirus L* proteins. The first 75 aa from the +1 frame shifted ORF are shown. Stop codons are depicted as *. Note that the L* proteins of the TMEV-GDVII strain and the SAFVs contain an ACG start codon. (0.07 MB PDF) [file ppat.1000416.s002.pdf]

A

```
SAFV-3 NL2007     -MA-------CKHGYP-LLCPLCTALDITPDGSFTLLFDNEWYPTDLLTVNLDDDVFYPLDT-----NMDWTDLPLIQDIVMEPQ
SAFV-2 UC1        -MA-------CKHGYP-LMCPLCTALDKTSDGLFTLLFDNEWYPTDLLTVDLEDEVFYPDDP-----HMEWTDLPLIQDIEMEPQ
SAFV-2 Can112051  -MA-------CKHGYP-LMCPLCTALDNTSDGLFTLLFDNEWYPTDLLTVDLEDEVFYPDDL-----HMEWTDLPLNQDIEMEPQ
SAFV-1            -MA-------CKHGYP-FLCPLCTAIDISADGSFALLFDNEWYPTDLLTVDLDDDVFHPPDC-----VMEWTDLPLIQDVLMEPQ
TMEV GDVII        -MA-------CKHGYP-DVCPICTAVDATPDFEYLLMADGEWFPTDLLCVDLDDDVFWPSDTSTQPQTMEWTDVPLVCDTVMEPQ
TMEV DA           -MA-------CKHGYP-DVCPICTAVDVTPGFEYLLLADGEWFPTDLLCVDLDDDVFWPSNSSNQSETMEWTDLPLVRDIVMEPQ
TRV1              MMA-------CIHGYP-SVCPICTAIDKSSDGMYLLLADNEWFPADLLTMDLDDDVFWPNDKSNVSETMDWTDLPFILDTVMEPQ
TRV NGS910        MMA-------CIHGYP-SVCPICTAIDKSSDGMYLLLADNEWFPADLLTMDLDDDVFWPNDESDVSETMDWTDLPFILDTIMEPQ
VHEV              -MA-------CKHGYP-DVCPICTAIDVTPGFEYLLLADGEWFPTDLLCVDLDDDVFWPSDSSNQSQTMEWTDIPLICDTVMEPQ
EMCV              -MATTMEQEICAHSMTFEECPKCSALQYR-NGFYLLKYDEEWYPEELLT-DGEDDVFDPDLD---------------MEVVFETQ
```

zinc finger | acidic domain | S/T-rich domain

B

```
SAFV-3 NL2007     TDIRFCALSALLWILLQTDHSLSCLIMSGIQPTF*
SAFV-2 UC1        TDIRLCALFALLSTKLRTDFSPFCSTMNGTQLTY*
SAFV-2 Can112051  TDIRLCALFALLSTTLRTDFSPFCSIMNGTQLTY*
SAFV-1            TDIRFCALFALLLTSLQMDLLLYYLTMNGTRLTSLLLTWTTTCFIPRI-----VLWNGLIYH*
TMEV DA           MDTQMCALFAQPLTLLPDLNICSWQTVNGSQRTFFVWTWTMTSSGLRTRAINLKQWNGLTYRSYAILSWNPRETP
TMEV GDVII        TDTQTCALFAQPLTLLPTLNICSWQTENGSLRTFFVWTWTMTSSGLRTRALNLKQWNGLMYRSYAILSWNPREMP
EMCV              LQPWNKRFVLIP*
```
