# Supplementary material for: Saffold Virus, a Human Theiler's-Like Cardiovirus, Is Ubiquitous and Causes Infection Early in Life
Source: PLoS Pathog. 2009 May 1;5(5):e1000416. doi: 10.1371/journal.ppat.1000416 (PMC2670511; doi:10.1371/journal.ppat.1000416)
Supplement: Table S1 — Nucleotide and amino acid (in parenthesis) identity of SAFV-3(NL2007) UTRs and proteins to other Theiloviruses from which full-length sequences or polyprotein coding regions are known. (0.09 MB PDF) [file ppat.1000416.s003.pdf]

**Table S1.** Nucleotide and amino acid (in parenthesis) identity of SAFV-3(NL2007) UTRs and proteins to other Theiloviruses from which full-length sequences or polyprotein coding regions are known.

| | 5'UTR | Leader | VP4 | VP2 | VP3 | VP1 | 2A | 2B | 2C | 3A | 3B | 3C | 3D | 3'UTR |
|---|---|---|---|---|---|---|---|---|---|---|---|---|---|---|
| SAFV-2 UC1 | 90 | 80 (83) | 82 (100) | 72 (77) | 71 (81) | 69 (73) | 85 (98) | 89 (99) | 88 (97) | 88 (100) | 82 (95) | 90 (96) | 89 (98) | 97 |
| SAFV-2 Can112051 | | 78 (82) | 82 (99) | 71 (77) | 71 (81) | 69 (73) | 85 (96) | 89 (98) | 88 (97) | 87 (99) | 82 (95) | 90 (96) | 88 (97) | |
| SAFV-1 | 91 | 79 (82) | 84 (99) | 71 (79) | 74 (85) | 64 (68) | 88 (98) | 89 (99) | 88 (97) | 87 (99) | 88 (100) | 89 (98) | 89 (98) | 97 |
| TMEV GDVII | 75 | 66 (68) | 70 (69) | 66 (70) | 70 (81) | 60 (55) | 63 (63) | 66 (62) | 72 (78) | 64 (66) | 63 (60) | 69 (77) | 70 (77) | 88 |
| TMEV DA | 75 | 66 (68) | 70 (69) | 67 (71) | 68 (79) | 58 (57) | 63 (61) | 66 (63) | 71 (79) | 66 (67) | 63 (60) | 69 (76) | 69 (77) | 87 |
| TRV-1 | 84 | 67 (68) | 69 (73) | 68 (73) | 70 (78) | 61 (53) | 65 (69) | 71 (74) | 77 (89) | 71 (78) | 67 (70) | 72 (81) | 73 (82) | 90 |
| TRV NGS910 | 84 | 66 (66) | 68 (74) | 67 (73) | 71 (79) | 61 (53) | 66 (69) | 72 (74) | 78 (90) | 70 (78) | 63 (70) | 72 (81) | 73 (82) | 89 |
| VHEV | 74 | 66 (67) | 70 (74) | 67 (71) | 71 (77) | 62 (55) | 64 (62) | 66 (62) | 71 (78) | 64 (69) | 65 (60) | 70 (77) | 69 (77) | 87 |
